# Supplementary material for: External Validation of Mortality Scores among High-Risk COVID-19 Patients: A Romanian Retrospective Study in the First Pandemic Year
Source: J Clin Med. 2022 Sep 24;11(19):5630. doi: 10.3390/jcm11195630 (PMC9573119; doi:10.3390/jcm11195630)
Supplement: Supplementary file 1 [file jcm-11-05630-s001.zip › jcm-1877587-SI.pdf]

Supplementary Tables

Supplementary Table S1. Performance of mortality scores as predictive tool for probability of death. All values were tested as cut-offs, resulting in a maximal accuracy and performance at 13 points (9–14—high risk) [17].

Cut-off	Accuracy	Youden's Index	Sensitivity	Specificity	Positive Predictive Value	Negative Predictive Value
4CM Score						
12	*66.7%*	*32.0%*	*63.9%*	*68.0%*	*50.0%*	*79.0%*
13	***71.1%***	***34.2%***	***55.1%***	***79.1%***	***56.9%***	***77.9%***
14	***71.9%***	***29.1%***	***42.4%***	***86.7%***	***61.5%***	***75.1%***
15	*71.7%*	*21.8%*	*28.5%*	*93.4%*	*68.2%*	*72.3%*

Supplementary Table S2. Performance of COVID-GRAM, COVIDAnalytics scores [21,22].

Cut-off	Accuracy	Youden's Index	Sensitivity	Specificity	Positive Predictive Value	Negative Predictive Value
COVID-GRAM						
65	*63.1%*	*32.3%*	*75.3%*	*57.0%*	*46.7%*	*82.2%*
70	***68.1%***	***38.0%***	***71.5%***	***66.5%***	***51.6%***	***82.4%***
75	*69.2%*	*36.4%*	*65.2%*	*71.2%*	*53.1%*	*80.4%*
80	*70.9%*	*36.1%*	*59.5%*	*76.6%*	*56.0%*	*79.1%*
85	*71.5%*	*33.2%*	*51.9%*	*81.3%*	*58.2%*	*77.2%*
90	***74.7%***	***33.5%***	***43.0%***	***90.5%***	***69.4%***	***76.1%***
95	*73.2%*	*23.4%*	*27.2%*	*96.2%*	*78.2%*	*72.6%*
COVIDAnalytics						
30	*64.5%*	*38.9%*	*83.6%*	*55.4%*	*47.4%*	*87.5%*
35	***69.2%***	***40.8%***	***73.7%***	***67.1%***	***51.9%***	***84.1%***
40	*69.0%*	*32.3%*	*57.9%*	*74.4%*	*52.1%*	*78.6%*
45	*70.9%*	*33.1%*	*53.9%*	*79.1%*	*55.4%*	*78.1%*
50	***74.6%***	***33.7%***	***44.7%***	***88.9%***	***66.0%***	***77.0%***
55	*74.1%*	*29.3%*	*37.5%*	*91.8%*	*68.7%*	*75.3%*
